# Listeners form average-based representations of individual voice identities

**DOI:** 10.1038/s41467-019-10295-w

**Published:** 2019-06-03

**Authors:** Nadine Lavan, Sarah Knight, Carolyn McGettigan

**Affiliations:** 1 Department of Speech, Hearing and Phonetic Sciences, University College London, London, WC1N 1PF UK; 2 Department of Psychology, Royal Holloway, University of London, Egham, TW20 0EX UK

**Keywords:** Psychology, Human behaviour

## Abstract

Models of voice perception propose that identities are encoded relative to an abstracted average or prototype. While there is some evidence for norm-based coding when learning to discriminate different voices, little is known about how the representation of an individual's voice identity is formed through variable exposure to that voice. In two experiments, we show evidence that participants form abstracted representations of individual voice identities based on averages, despite having never been exposed to these averages during learning. We created 3 perceptually distinct voice identities, fully controlling their within-person variability. Listeners first learned to recognise these identities based on ring-shaped distributions located around the perimeter of within-person voice spaces – crucially, these distributions were missing their centres. At test, listeners’ accuracy for old/new judgements was higher for stimuli located on an untrained distribution nested around the centre of each ring-shaped distribution compared to stimuli on the trained ring-shaped distribution.

## Abstract (web summary)

People can learn to identify a person based on their voice, despite variation in their voice. Here, the authors show that this ability relies on a statistical abstraction mechanism during which people form average-based representations of voices, even without prior exposure to the average.

## Introduction

The question of how we learn and represent person identity has long been debated. For voices, a prominent view proposes that different identities are encoded on a multidimensional voice space in relation to a prototype voice. In such a view, the prototype is thought to be a representation of either an average voice, or a very frequently encountered voice^1–5^; see refs. ^6,7^ for reviews. Within such voice spaces, the location of an individual voice relative to the prototype has perceptual implications: Voices that are further away from this between-person prototype are more perceptually distinctive (see refs. ^4,5,8^; for faces see ref. ^9^) and an identity’s distance to the prototypical voice affects how well it is remembered and recognised. For example, some studies find evidence that distinctive voices are more reliably remembered^8,10^, while others find evidence for the opposite pattern, with recognition remaining more reliable after a delay for prototypical voices^8^.

Studies exploring prototype- or norm-based coding tend to conceptualise different voice identities as single points in the voice space. These studies therefore focus on between-person variability, and on how listeners discriminate between different  identities. While telling different voices apart is one important aspect of how we process voice identity information, the fact that individual voices are highly flexible is often neglected in these studies. The acoustic and perceptual properties of a individual’s voice can vary dramatically depending on the type of speech or vocalisations produced (shouting vs. reading aloud vs. joking with friends vs. whispering^11^). Listeners are therefore not only required to encode how different people’s voices differ from each other; they also need to represent how the different and variable instances of a single person’s voice belong to the same identity. This can be a challenging task: it has been shown that listeners who are unfamiliar with a voice struggle to match, or “tell together”, naturally-varying instances of speech produced by the same person^12^. Similarly, accurately perceiving identity across different vocalisations (laughter vs. vowels) is challenging for familiar and unfamiliar voices alike^13,14^. By failing to explicitly account for how within-person variability impacts on the learning and perception of voices, current models of voice identity perception remain incomplete and underspecified.

While norm-based coding and the extraction of summary statistics are at the centre of many models of identity perception, only a relatively small number of empirical studies have provided direct evidence of such a mechanism for faces and voices. In these studies, participants were presented with the voices or faces of familiar (or familiarised) identities: some of the stimuli were unmanipulated voice recordings/images of faces, while others were averages derived from different numbers of original stimuli. This approach assumes that the nature of a within-person representation is approximated by averaged stimuli with the rationale that averaging preserves the diagnostic information about the person’s identity while reducing non-diagnostic variability introduced by external factors (competing noises, quality of the signal transmitted, etc.) across different instances^15–17^. A study of voice processing by Fontaine and colleagues^17^ has explored how recognition accuracy and associated reaction times are affected by averaging familiar or newly trained-to-familiar voices. For familiar celebrity voices, the authors indeed found that the higher the number of recordings contributing to an average stimulus, the higher the explicit identity recognition accuracy for these averages, with reaction times correspondingly decreasing. The opposite effect was, however, reported for the trained-to-familiar voices: here, recognition accuracy decreased with increasing averageness, while reaction times remained stable. Why the effects did not replicate across the experiments is uncertain. Additional evidence for averageness aiding identity perception comes from a study using familiar (celebrity) faces as stimuli: here, reaction times decreased when the number of individual images used to create the average was increased^16^. Thus, averages may under certain circumstances form a meaningful part of how familiar voices (and faces) are represented.

Evidence for how an average may become a meaningful part of a representation can be found in studies of ensemble coding using sets of faces. It has been shown that viewers routinely extract summary statistics from sets of faces, such as the average emotional expression of faces^18–20^, and gender^19^. For identity processing, there is evidence that average identity information is represented for unfamiliar as well as familiar faces, and for sets of images of a single identity as well as multiple identities presented simultaneously or sequentially^21–23^. Studies specifically probing within-identity processing in faces have shown that when viewers are briefly presented with a small number of variable pictures of a single identity, (previously unseen) average pictures derived from all presented pictures are labelled as previously seen with the same frequency as the individual pictures actually seen before^23^. Notably, viewers also seem to retain information about the specific exemplars viewed alongside these averages^22,23^. In the auditory modality, there is evidence that listeners extract summary statistics from heard stimuli, such as the mean frequency of small sets of pure tones^24,25^ and sound textures^26,27^. To our knowledge, however, no study has directly probed whether summary statistics are extracted for voice identities.

The extraction of summary statistics from sets of stimuli seems to be commonplace in perception, but what is its purpose? Encoding abstracted statistical information compared to high-resolution and possibly redundant exemplar-based information is computationally efficient^28^. While the extraction of summary statistics has therefore been proposed as a candidate mechanism for how representations of individual face identities are formed^23^, the studies on ensemble coding reviewed above rely on the brief presentation of a relatively small set of stimuli and do not entail any formal training or familiarisation procedure. It is thus unclear whether and how the extraction of summary statistics from faces using these paradigms extends to (1) voices and (2) paradigms that focus on training listeners to learn to recognise different voice identities and thus form representations of these voices.

Here, we ask whether listeners abstract summary statistics of salient acoustic cues from a distribution of voice samples when learning to recognise individual voice identities. We show that listeners more accurately recognise newly learned voice identities from previously unheard acoustically average stimuli than from stimuli whose acoustic properties map directly onto distributions heard during training. This finding suggests that listeners do indeed abstract summary statistics when learning new voice identities, highlighting averages as a potentially meaningful part of mental representations of voice identities.

## Results

### Abstraction of averages for the learned identities

**Fig. 1:**
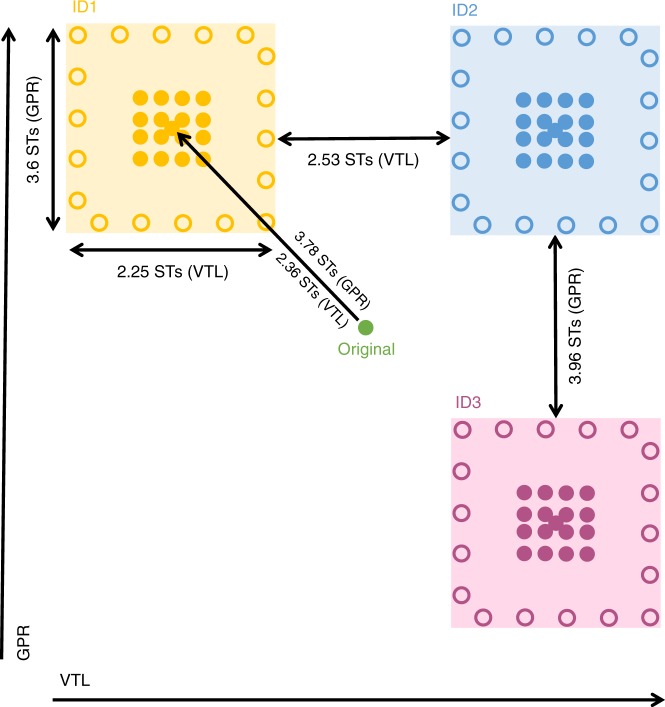
Illustration of the 2D voice space and the voice identities we created. VTL = vocal tract length, GPR = glottal pulse rate, ST = semitones. Shaded squares show the nominal within-person voice spaces for the 3 identities created from a sentence produced by one original talker (green). Empty dots arranged around the perimeter of the individual voice spaces show the locations of stimuli from the ring-shaped distribution used during the learning phase; filled dots show the locations of the stimuli forming the centre distribution introduced at test. Two sets of identities were created in this way, each based on a different original speaker’s voice. One set of identities was used during the learning phase; the second set was introduced to listeners as distractor identities at the old/new recognition task, which took place after the learning phase. Assignment of the two sets of identities as trained and distractor identities was counterbalanced across the participant sample. Please see the Methods section and Supplementary Note 1 for more details on the arrangement and assignment of training and distractor stimuli

To investigate whether listeners abstract summary statistics—specifically averages—when learning new voice identities, we created a two-dimensional acoustic voice space. Within this voice space, we were able to fully describe and control the degree and nature of the variability in our stimuli and thus specify stimulus distributions and their summary statistics. The voice space was defined by variation in glottal pulse rate (GPR) in one dimension, and variation in apparent vocal tract length (VTL) in the other dimension (Fig. 1). By systematically manipulating the GPR and VTL of one original speaker’s voice within our voice space, we were able to create distinct voice identities (e.g. refs. ^29,30^). Each identity included acoustic variation across exemplars that was perceptually acceptable for a single talker (see Methods)—these variations therefore created a within-person voice space for each identity. In two experiments, listeners learned to recognise these voice identities based on ring-shaped distributions tracing the perimeter of the within-person voice spaces over the course of two brief learning tasks. These ring-shaped distributions were missing their centres, thus never exposing listeners to the acoustic average during the learning phase. After the learning phase, listeners provided old/new judgements for stimuli located on both the previously trained ring-shaped distribution and, crucially, a previously untrained centre distribution nested around the acoustic average of each identity’s ring-shaped distribution (Fig. 1). Distractor identities for the task—the “new” voices—were created in an identical way to the learned identities but based on a different original speaker. Their overall voice quality was therefore similar to the learned voices, increasing task difficulty at test. The stimuli used for the training and test phases were based on “delexicalised”, read sentences, where every syllable was articulated as “na” to reduce interference from lexical information in the stimuli, while retaining many other characteristics of the voice, such as pitch, intonation, speech rate and general voice quality.

If acoustic averages are abstracted when forming representations of new individual voice identities, then accuracy on an old/new recognition task for learned voices should be the same or better for untrained stimuli located around the geometric centre of the distribution than for the trained stimuli located on the ring-shaped distribution. Similarly, accuracy should increase for stimuli that are acoustically closer to the centre or average. If averages are not abstracted, however, then accuracy on the old/new judgement task should be worse for the untrained stimuli in the centre of each within-person voice space compared to accuracy for trained stimuli located on the ring-shaped distribution. This should also be reflected in decreasing accuracy with increasing acoustic proximity to the centre of the ring-shaped distribution (i.e. acoustic average).

40 participants took part in Experiment 1. To assess whether averages are abstracted during voice identity learning, we analysed listeners’ accuracy on the post-learning old/new recognition task, for the learned identities only (i.e. not taking the data from the distractor identities into account). We ran a binomial intercept-only generalised linear mixed model (GLMM) using *lme4*^31^ in the *R* environment^32^. Distribution type (ring-shaped or centre) was included as a fixed effect. We also included a nested random effects structure accounting for different identities, versions stimulus sets and individual stimuli. The model specification is detailed in the formula below. We diverged from the preregistered analyses across all analyses reported in this paper in terms of the random effects structure, to avoid singular fits.

1 $${\mathrm{glmer}}({\mathrm{Accuracy}} \sim {\mathrm{distribution}} \\ 	+ (1|{\mathrm{participants}}) + (1|{\mathrm{identity}} \, {\mathrm{by}} \, {\mathrm{speaker}} \\ 	 / {\mathrm{versions}} \, {\mathrm{of}} \, {\mathrm{stimulus}} \, {\mathrm{sets}} / {\mathrm{stimulus}}), {\mathrm{family}} \\ 	= {{\mbox{"}}{\rm binomial} {\mbox{"}}}) $$

**Fig. 2:**
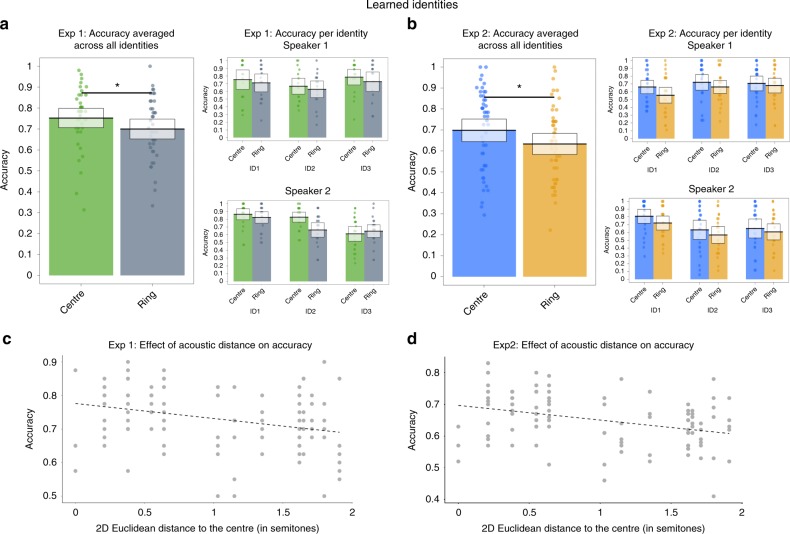
Summary of old/new recognition performance at test for learned identities for Experiment 1 (left-hand panels) and Experiment 2 (right-hand panels). **a, b** Accuracy for the two distributions (centre and ring-shaped) is plotted averaged across all identities and both speakers, and broken down by identity and speaker. Boxes show the 95% confidence intervals, dots indicate the mean accuracy per participant. **c**, **d** Scatterplots plotting each stimulus’ 2D Euclidean distance in semitones to the centre of each identity’s nominal voice space against the average performance for each location on the voice space. Asterisks denote a significant effect of distribution type (centre vs. ring-shaped) on accuracy as determined via likelihood ratio tests (see Results). Source data are provided as a Source Data file

Statistical significance was established via likelihood ratio tests contrasting the full model including the fixed effect plus the random effects with a null model that did not include the fixed effect. Coefficients were transformed into probabilities (probability = exp[coeff]/[1 + exp[coeff]]) for ease of interpretation. These confirmatory analyses showed that the distribution type had an effect on accuracy (coefficient of −0.32, SE = 0.09; probability = 0.42) and the comparison of the full and null model was significant (*χ*^2^[1] = 11.96, *p* = 0.001). Accuracy was significantly higher for stimuli from the previously unheard centre distribution that overlaps with the acoustic average compared to stimuli from the trained, ring-shaped distribution (Fig. 2a on the left). This numeric trend was apparent for 5 out of 6 trained identities (Fig. 2a on the right).

To assess whether there is a relationship between accuracy and acoustic distance to the centre of each identity’s voice space, we ran a complementary binomial GLMM. Here, the 2D Euclidean distance from each stimulus to the centre of each identity’s voice space was included as a fixed effect. We again included a nested random effects structure accounting for different identities, versions of stimulus sets and individual stimuli.

2 $$	{\mathrm{glmer}} ({\mathrm{Accuracy}} \sim {\mathrm{2D}}\,{\mathrm{Euclidean}}\,{\mathrm{distance}}\,{\mathrm{to}}\,{\mathrm{the}}\, \\ 	{\mathrm{centre}} + ({\mathrm{1}}|{\mathrm{participants}}) \\ 	+ ({\mathrm{1}} |{\mathrm{identity}}\,{\mathrm{by}}\,{\mathrm{speaker}}/{\mathrm{versions}}\,{\mathrm{of}}\,{\mathrm{stimulus}}\, \\ 	 {\mathrm{sets}}/{\mathrm{stimulus}}),{\mathrm{family}} = {{\mbox{"}}{\rm binomial} {\mbox{"}}})$$

These models confirmed that there was a negative relationship between the accuracy and distance to the centre (coefficient of −0.18, SE = 0.08; probability = 0.46) and the comparison of the full and null model was significant (*χ*^2^[1] = 5.55, *p* = 0.018). Thus, accuracy increases the closer a stimulus is to the previously unheard centre (acoustic average) of the trained distribution (Fig. 2c). We furthermore conducted exploratory analyses assessing whether the reported effects of acoustic distance to the centre hold for each acoustic dimension independently. Models were identical to the ones reported above but included the Euclidean distance in semitones calculated for each dimension separately. For both GPR and VTL dimensions, accuracy increased the closer an item was to the centre of the training distribution. This effect was, however, only significant for acoustic distances computed for VTL (VTL: coefficient = −0.41, SE = 0.12; probability = 0.40; *χ*^2^[1] = 10.98, *p* = 0.001, GPR: coefficient = −0.10, SE = 0.08; probability = 0.48; *χ*^*2*^[1] = 1.73, *p* = 0.189).

The results of Experiment 1 thus suggest that averages are indeed abstracted when learning new voice identities and may form a meaningful part of voice identity representations: accuracy for an old/new recognition task was higher for the previously untrained geometric centre of the training distribution than for the training distribution itself. This effect was also apparent when modelling the location on the within-person voice space in terms of the distance between a stimulus’ acoustic properties and the acoustic centre of an identity’s voice space: accuracy was higher for stimuli closer to the unheard centre or acoustic average.

In Experiment 2, we replicated and extended our findings to more naturalistic linguistic stimuli: using the same experimental design, we trained listeners on recordings of full sentences with their linguistic content intact, instead of using delexicalised sentences. However, the delexicalised stimuli were still used at test. By training listeners on sentences including linguistic content but testing them on delexicalised sentences, we additionally required listeners in this experiment to generalise information about the learned identities across different types of stimuli from training to test.

Fifty listeners took part in Experiment 2. Data were analysed in the same way as for Experiment 1: in a confirmatory analysis, we first contrasted accuracy for the centre distribution vs. the ring-shaped distribution, for the learned identities only. These models confirmed that the distribution type had an effect on accuracy (coefficient of −0.35, SE = 0.07; probability = 0.41; *χ*^2^[1] = 25.34, *p* < 0.001). We thus replicated the effect reported in Experiment 1 showing that accuracy is higher for stimuli from the previously unheard centre distribution compared to stimuli from the trained, ring-shaped distribution (Fig. 2b on the left). This numeric trend was apparent for all 6 trained identities (Fig. 2b, right).

We tested whether accuracy was related to the acoustic distance to the centre in a second confirmatory analysis. These models again confirmed that there was a negative relationship between the two measures (coefficient of −0.25, SE = 0.06; probability = 0.44; *χ*^2^[1] = 19.85, *p* < 0.001). Thus, we replicated our findings from Experiment 1, showing that accuracy increases the closer a stimulus is to the centre of the trained distribution (Fig. 2d). We then assessed in an exploratory analysis whether the effect of acoustic distance holds for both GPR and VTL dimensions individually. Models were identical to the ones reported for Experiment 1. We replicated our findings showing that accuracy increased the closer an item was to the centre of the training distribution for each of the dimensions (VTL: coefficient = −0.34, SE = 0.09; probability = 0.42; *χ*^2^[1] = 13.40, *p* < 0.001, GPR: coefficient = −0.20, *SE* = 0.06; probability = 0.45; *χ*^2^[1] = 11.77, *p* < 0.001). This result differs from Experiment 1 where only the model coding for the VTL dimension showed a significant effect.

### Effects of voice space location on the distractor identities

In the old/new recognition task, half of the trials included stimuli from the previously learned identities (which are analysed in the previous paragraphs), while the other half included stimuli from a matched set of distractor identities—the “new” voice identities. The stimulus distributions for the distractor identities were created in the same way as the distributions for learned identities (see Methods and Figure 1): Crucially, the original speaker used to create the distractor voices was however a different speaker to the one used to create the learned identities. We aligned the salient acoustic properties (GPR and VTL) for learned and distractor identities, while all other acoustic differences between original speakers were allowed to vary freely. Consequently, ring-shaped and centre distributions were largely overlapping in their acoustic properties across learned and distractor voices (see Methods).

**Fig. 3:**
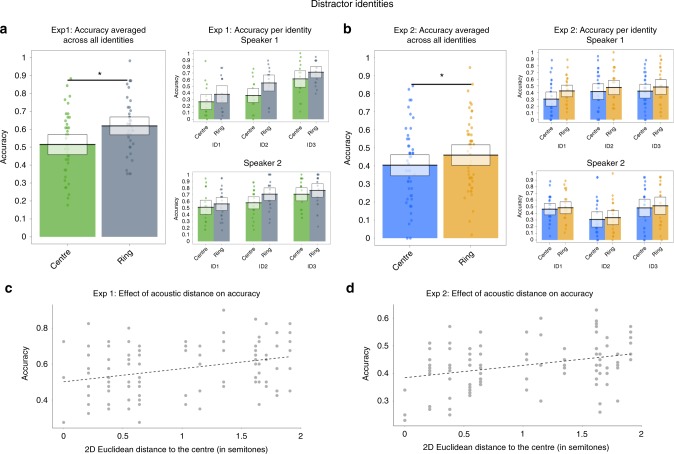
Summary of old/new recognition performance for distractor identities for Experiment 1 (left-hand panels) and Experiment 2 (right-hand panels). **a**, **b** Accuracy for the two distributions (centre and ring-shaped) is plotted averaged across all identities and both speakers, and broken down by identity and speaker. Boxes show the 95% confidence intervals, dots indicate the mean accuracy per participant. **c**, **d** Scatterplots plotting each stimulus’ 2D Euclidean distance in semitones to the centre of each identity’s nominal voice space against the average performance for each location on the voice space. Asterisks denote a significant effect of distribution type (centre vs. ring-shaped) on accuracy as determined via likelihood ratio tests (see Results). Source data are provided as a Source Data file

In exploratory analyses, we investigated whether any relationship between accuracy in the old/new recognition task and the type of distribution (ring-shaped vs. centre) existed for the distractor identities as well as for the learned identities, given the acoustic overlap between the two identity sets. For this purpose, we ran a GLMM analysis on accuracy, which was identical in its structure to the one run for the learned identities: it included the distribution type as a fixed effect and a nested random effects structure including random effects for the different identities, versions of stimulus set and stimuli. For Experiment 1, these analyses showed that the distribution type on the voice space had an effect on accuracy (coefficient of 0.52, SE = 0.08, probability = 0.63; *χ*^2^[1] = 40.00, *p* < 0.001). The direction of the effect was, however, the opposite of that for learned identities (Fig. 3a): Accuracy was higher for stimuli on the ring-shaped distribution and lower for the centre distribution, a trend that held for all 6 identities.

We further explored whether there was a relationship between accuracy and acoustic distance to the centre of each distractor identity’s voice space in Experiment 1, mirroring the GLMMs specified for the learned identities. These exploratory analyses also showed the opposite effect to what we observed for the learned identities: There was a positive relationship between accuracy and distance to the centre (coefficient of 0.45, SE = 0.07; probability = 0.61; *χ*^2^[1] = 43.783, *p* < 0.001). Accuracy thus decreased the closer a stimulus was to the centre of the trained ring-shaped distribution (see Fig. 3b). This effect also held when considering VTL and GPR independently (VTL: coefficient of 0.58, SE = 0.11; probability = 0.64; *χ*^2^[1] = 26.71, *p* < 0.001; GPR: coefficient of 0.39, SE = 0.07; probability = 0.60; *χ*^2^[1] = 31.03, *p* < 0.001).

We conducted the same analyses for the data from the distractor identities in Experiment 2 and found again that the type of distribution had an effect on accuracy (coefficient of 0.20, SE = 0.06; probability = 0.55; *χ*^2^[1] = 9.75, *p* = 0.001, see Fig. 3c). The trend of better performance for the ring-shaped distribution held for all 6 identities. Similarly, we replicated the finding showing an effect of acoustic distance to the centre on accuracy when considering distance in two dimensions (coefficient of 0.19, SE = 0.05; *χ*^2^[1] = 12.60, *p* < 0.001, see Fig. 3d) and also when considering each dimension individually (VTL: coefficient of 0.32, SE = 0.09; probability = 0.55; *χ*^2^[1] = 13.71, *p* < 0.001; GPR: coefficient of 0.15, SE = 0.05; probability = 0.54; *χ*^2^[1] = 8.02, *p* = 0.005). Across the two experiments and the two types of analyses, we therefore consistently found the opposite pattern of results for distractor identities to what was found in the confirmatory analyses for the learned identities.

## Discussion

We investigated whether averages are abstracted during the learning of new individual voice identities. Across two experiments, participants learned to recognise 3 new identities based on variable voice stimuli forming ring-shaped distributions on a two-dimensional voice space. Accuracy for a subsequent old/new recognition task was higher for an untrained distribution of stimuli grouped around the centre—the acoustic average—of the learned distribution than for stimuli located on the previously trained ring-shaped distribution. The current studies are therefore direct empirical demonstrations that participants indeed extract averages when forming abstracted representations of individual voice identities and appear to use them during subsequent recognition, despite having never been exposed to these averages during learning. Since our participants were not given any specific instructions on how to learn the different identities and will have had limited insight into the properties of the different stimulus distributions, it is likely that the extraction of summary statistics occurred automatically during learning. This is in line with previous research that has shown that listeners automatically learn about the relationships of acoustic properties of artificially created complex sounds that vary systematically along two dimensions^28^. All effects held across two independent participant samples and two types of training stimuli: in Experiment 1, delexicalized sentences were used while in Experiment 2 we used sentences that included full linguistic content. In comparison to Experiment 1, overall performance was lower in Experiment 2 (e.g. 69% for the centre distribution vs. 76% in Experiment 1): this most likely reflects the cost of additional generalisation across stimulus types from training (sentences including lexical content) to test (delexicalized sentences) in Experiment 2.

Models of voice perception have proposed that an average voice may form a perceptually meaningful prototype, in relation to which individual identities are encoded. In the context of the extensive within-person variability of human voices, it is reasonable to assume that a similar process is at work when forming a representation of a new voice identity based on variable signals^11^. Our findings confirm this proposal and can thus be interpreted as preliminary evidence for the existence of abstracted, norm-based within-person representations of voice identity as a result of being exposed to a variable signal. Our results additionally could be interpreted as evidence against exemplar-based models of identity coding, which are frequently used as an alternative to norm-based coding models (e.g. ref. ^9^ for faces). Exemplar-based models would predict the highest accuracy for stimuli that are acoustically closer to what was previously heard. Although we note that we define exemplars as specific locations in a voice space, as opposed to specific stimuli (i.e. we did not repeat the same recordings across learning and test), an exemplar-based account should still predict lower accuracy at test for previously unheard locations in the centre distribution. We note that while an exemplar model of representation would have predicted the opposite to the observed pattern of results, listeners may still have retained representation of the previously heard exemplars, since overall accuracy for stimuli from both distributions was relatively high.

As part of exploratory analyses, we found that in both experiments, accuracy for the distractor identities exhibited the opposite pattern of results to the learned identities: accuracy (i.e. correctly labelling a distractor identity as “new”) was *higher* for stimuli located on the ring-shaped distribution compared to stimuli located on the centre distribution. Similarly, accuracy *decreased* the closer stimuli were to the centre (whether considering both acoustic properties together, or modelling them individually). How these exploratory findings should be interpreted remains unclear: it may be that these effects arose due to the overlap in the salient acoustic properties (GPR and VTL) between distractor and learned identities, although we have little insight into how the acoustic cues shaped listeners’ responses to the previously unheard distractor identities. For further speculative interpretations of these findings as evidence for average-based coding or in relation to possible task-related effects, please see Supplementary Note 2.

How can our findings be specifically integrated into existing models of voice perception? Given the flexibility and natural variability of our voices, we are likely to not only encode abstracted representations of how different voices relate to a single underlying prototype and to each other. We also need to form representations of how different variable exemplars of the same voice go together—that is, we must form a within-person representation of a voice. For the current studies, we report that listeners extract identity-specific summary statistics when learning new voice identities. We propose that this process may underpin how representations of voice identities are formed (see also ref. ^23^ for faces). If voice identity perception thus involves both within- and across-identity representations, the question arises of how these two representations or processes interact. First, there appears to be a logical hierarchy to the order in which within- and between-person representations are formed when learning a new voice. Upon first exposure to a new voice identity, it can be rapidly established where this identity falls on the between-person voice space^2^: this voice space is already populated with a large number of familiar voices that we have encoded throughout our lives. These familiar voices have already mapped out and defined the dimensions and boundaries of the between-person voice space and have crucially created a prototypical voice that is used as an anchor for between-person identity coding (see ref. ^6^ for a review). Being exposed to a single exemplar may thus be sufficient to quickly assess with reasonable accuracy that the voice just heard is an unknown one. This hypothesis is supported by the finding that listeners who are unfamiliar with a set of voices have little trouble telling these voices apart with high accuracy, with performance comparable to that of listeners who are already familiar with the voices^12^. Note, however, that false alarms (i.e. labelling unfamiliar voices as familiar) have also been reported in the literature^33^. When first encountering a new voice, however, by definition no within-person representation or prototype for this voice exists until multiple exemplars have been heard. Thus, after establishing that a voice is indeed unknown, a within-person representation and voice space must then be mapped out for it to become a truly familiar voice. Our results suggest that this may take place through extracting summary statistics that will eventually, through increasing exposure to this voice, help to form a stable within-person prototype^6^.

We used a method that allowed us to create distinctive voice identities featuring within-person variability, with tight control over the acoustic and perceptual properties of the stimuli. This control was essential to create distributions of stimuli with known properties and quantify their statistical averages. Through this process, ecological validity was sacrificed: we introduced variability for each identity by changing only two perceptually salient acoustic properties of the stimuli (GPR and VTL). While GPR changes significantly in everyday voice use, there are no good estimates of whether and how VTL might change^29,30^. Natural variability of a voice is not two-dimensional but highly multi-dimensional, such that any number of acoustic features will be modified by simple changes in speaking style (conversational speech vs. read speech) or interlocutor (infant-directed speech vs. speaking with an adult^11^). One promising method that can introduce higher-dimensional manipulations of variability may, for example, be the use of morphing techniques through which continua between two voices can be created^3^. It has furthermore been demonstrated that within-person variability may be at least partitially idiosyncratic, that is, specific to an individual and therefore potentially diagnostic when perceiving identity as opposed to being noise (ref. ^34,35^ for faces): the identities created in the current study each varied in terms of the absolute GPR and VTL, but *how* identities varied was identical. Finally, the variability of specific acoustic properties in everyday vocal behaviour is likely to follow a broadly normal distribution (ref. ^36^ for GPR), making our training distribution highly artificial because all exemplars were presented with equal frequency. This type of training distribution was, however, specifically chosen to avoid the confounding effects of frequency of exposure: for normally distributed variability, the most average samples will also be the ones that are heard most frequently (ref. ^9^ for a discussion for faces).

 Our study opens up exciting avenues for future research to further elucidate the mechanisms underpinning the reported effects. For example, future research will need to explore whether  listeners form pure mathematical averages, or whether they take the precise distribution of exemplars into account to form weighted-average, prototypical representations. This could be tested explicitly by examining how outlier exemplars influence the properties of learned averages. Further, it remains to be determined whether and information about the variability is abstracted and/or encoded when forming representations. Another key challenge will be to investigate whether the averaging mechanisms suggested by our current data are comparable to those at work in richer, more naturalistic learning environments and over longer periods of exposure and learning: how much exposure is needed to observe these effects, and do they depend on the degree of within-person variability encoded during training? Since we can see significant averaging effects already after a minimal retention span during which exemplar-based memory traces should still be available, we might predict that the average-based abstraction becomes yet stronger over longer retention periods during which representation of specific exemplars might progressively fade. Finally, the extraction of summary statistics can be observed in the visual as well as in the auditory modality, both in the context of ensemble coding as a result of being exposed to sets of stimuli for a very short amount of time and—as this study has also shown—in the context of longer learning or training paradigms. Whether these findings rely on the same underlying mechanisms across modalities, or whether they are unrelated, remains an open question. Despite these open questions,  our study  is a first promising investigation into the mechanisms used to form within-person representation of voices, and highlights once again the importance of accounting for within-person variability in models of voice perception.

## Methods

### Participants

In Experiment 1, 44 participants were tested online using Gorilla (gorilla.sc/about^37^). Participants were recruited via Prolific (prolific.ac) and were reimbursed for their time. The study was approved by the ethics committee at Royal Holloway, University of London and researchers complied with all relevant regulations for work with human participants. All participants were aged between 18 and 40 years, were native speakers of English, had no reported hearing difficulties and had an approval rate over 90% on Prolific. No participant had taken part in any pilot or validation studies associated with this project. Four participants were excluded from this data set: 1 participant failed to give the correct response for more than 20% of vigilance trials (see Methods) and 3 participants did not perform significantly better than chance (±95% confidence intervals) for the last 15 trials of Training 2 (see Procedure). The final participant sample thus included 40 participants (mean age: 29.37 years, SD = 6.05 years; 19 females). This sample size was determined to be adequate through a power analysis based on a small pilot study (*N* = 15) using a similar paradigm.

In Experiment 2, 56 participants were also tested online using Gorilla^37^. Participants were recruited via Prolific (prolific.ac) and were reimbursed for their time. The study was approved by the ethics committee at the Department of Speech, Hearing and Phonetic Sciences at University College London and researchers complied with all relevant regulations for work with human participants. Eligibility criteria were the same as for Experiment 1. No participant had taken part in any pilot, validation studies associated with this project, or Experiment 1. Six participants were excluded from the sample as they did not perform significantly better than chance (±95% confidence intervals) for the last 15 trials of Training 2. The final participant sample thus included 50 participants (mean age: 29.57 years, SD = 6.39 years; 32 females, 1 other). This sample size was determine to be adeqaute based on the data from Experiment 1.

### Speakers and voice recordings

We recorded two male speakers (Speaker 1 and Speaker 2) of Standard Southern British English producing 69 sentences from the Bamford–Kowal–Bench corpus^38^ (e.g. “The clown had a funny face”). In addition to reading the sentences out with their linguistic content intact (see training stimuli in Experiment 2), speakers were instructed to replace every original syllable with “na”. These recordings formed the basis of the training and test voices used later in Experiment 1. Sentences were recorded in a sound-attenuated booth using a Røde NT1A microphone. All sentences were saved as WAV files, normed across stimuli to match the median pitch of each speaker, and finally normed for RMS amplitude. Stimuli were converted into MP3 format for use on the online testing platform. In the study, one of the speakers was used as the basis for the training voice identities while stimuli based on the second speaker were introduced at the test phase as distractor identities. We counterbalanced the assignment of speakers to training and test phases across participants.

### Creating distinct voice identities

Based on each originally recorded speaker’s sentences, we created 4 perceptually distinct voice identities by shifting the GPR (related to the fundamental frequency and voice pitch perception) and VTL (related to voice timbre perception) in Praat^39^ with the methods used by Darwin, Brungart and Simpson^40^, thus creating a two-dimensional voice space (Fig. 1). GPR and VTL have previously been shown to be the most salient cues for identity perception^2,29,30,40,41^, allowing us to create perceptually distinct voice identities that differed only in two known acoustic properties (GPR and VTL). We were thus able to fully control, quantify and reproduce the properties and variability of the stimuli on which listeners were trained and tested. Furthermore, all stimuli were manipulated using the same procedure, thus minimising potential confounds introduced by comparing manipulated and unmanipulated stimuli.

In our voice space, changes in GPR and VTL were perceptually equated, where a 1 semitone change in VTL corresponded to a 1.6 semitone change in GPR^29^. The centres of each the 4 identities were displaced from the original voice by 3.78 semitones for GPR and 2.36 semitones for VTL. Around these centres, we created nominal within-person voice spaces to simulate some of the natural within-person variability encountered in human voices (Fig. 1)^11^. Each within-person voice space had a range of 2.25 semitones for VTL and 3.6 semitones for GPR; thus, stimuli furthest away from each other within each voice space was perceived ambiguous to unfamiliar listeners as to whether they could have been produced by the same speaker or not, as assessed via a speaker discrimination task (Supplementary Fig. 1 and ref. ^29^). After reviewing the perceptual qualities of the 4 voice identities, one voice identity (low GPR, short VTL; missing in the left-hand bottom corner in Fig. 1) was excluded due to this identity being perceived as sounding unnatural during perceptual piloting of the identities derived from one of the speakers, with stimuli being prone to distortions introduced by the acoustic manipulation. For information on the perceptual properties of the voice identities, please see Supplementary Note 1.

### Stimuli

For the main experiments, we created two distributions of locations from each identity’s within-person voice space to allow us to test whether averages are formed when learning voice identities. These distributions are illustrated in Fig. 1. For the learning phase of the study, where participants learned to recognise the 3 different identities, we created a ring-shaped distribution per identity (18 locations × 3 identities). During learning, listeners were therefore never exposed to the average of the salient acoustic features for the individual identities. At test, we introduced a second type of distribution to participants (see “Procedure”). This type of distribution is nested within each ring-shaped training distribution and is located on and around the centre (and thus the acoustic average) of each nominal within-person voice space (17 locations × 3 identities). For the stimuli used in the main study, the different voice space locations were based on different underlying sentences (see Supplementary Note 1); sentences used at test had not been used in the training stimulus sets. Individual sentences were repeated 6 times each during training and 4–5 times during test. To minimise stimulus-specific effects, we furthermore created two versions of stimulus sets. For the two versions of stimuli sets, the combinations of sentences and locations on the voice space were shuffled; for example: if Sentence 1 was manipulated to correspond to the centre location of the voice space of ID1 in Stimulus Set 1, this same sentence could be manipulated to correspond to a location on the ring-shaped distribution for ID1 in Stimulus Set 2. These versions of stimulus sets were counterbalanced across participants.

### Procedure

For both experiments, participants were first provided with an information sheet and provided consent to take part in the study. They then completed a headphones screening^42^ before completing two brief learning phases (Training 1 and Training 2). For Training 1, participants were presented with stimuli covering all 18 locations on the ring-shaped training distribution while a name (Peter, James or Michael) was presented on the screen. These presentations were blocked by identity and the order of identities was randomised across participants. Participants were instructed to listen attentively while memorising the different identities and their names. No responses were collected during this training phase. For Training 2, participants were presented with the same stimuli again in fully randomised order and were asked to complete a 3-way forced choice recognition task (“Is this Michael, James or Peter?”) with audio–visual feedback on whether their response was correct or not. Both learning phases were self-timed and lasted on average between 5 and 8 min in total. Performance for the final 15 trials of Training 2 were used as an index to track whether listeners had learned to recognise the two identities. These data showed listeners were able to correctly identify the 3 voice identities with high accuracy towards the end of the learning phase (mean accuracy = 88.6%, SD = 9.9%; chance level = 33%).

After this learning phase, participants completed an old/new judgement task for the test phase. This task included stimuli from both the trained ring-shaped distributions (18 locations × 3 identities × 2 speakers (learned, distractor)) and the centre-distribution of the 3 identities (17 locations × 3 identities × 2 speakers (learned, distractor)). Listeners were presented with 6 nominal artificially created  identities: 3 learned identities based on one original speaker and 3 distractor identities based on the other original speaker. To increase the task difficulty, the GPR/F0 median was matched for the distractor identities to the GPR/F0 median for the learned identities. This was achieved by using the “change pitch” function in the Praat Vocal Toolkit^43^ to match the recordings of the original speaker used for the distractor identities to the median pitch of the original speaker used for the trained identities. The distractor identities were created afterwards. VTL was not explicitly aligned across speakers: VTL estimations based on 5 of the unmanipulated recordings however showed that Speakers 1 and 2 were well-matched in their VTLs (Speaker 1: 15.4 cm, Speaker 2: 15.6 cm; for methods see ref. ^44^). We determined that this difference of 0.2 cm in VTL broadly corresponded to around 2 manipulation steps within our voice space (each being 0.23 semitones for VTL). We, however, note that these estimates should be treated with caution, since small differences in vowel quality and formant measurement errors may have influenced these VTL estimates across speakers. Thus the learned and distractor identities overlapped fully on the GPR dimension and partially overlapped on the VTL dimension. Participants additionally completed 20 vigilance trials: here, a computer-generated voice instructed listeners to either respond with “old voice” or “new voice” (see exclusion criteria). The order of presentation was fully randomised across participants. The task was self-paced and participants were advised to complete the task in one sitting, without taking breaks. It took participants between 15 and 20 min to complete the full experiment.

### Reporting summary

Further information on research design is available in the Nature Research Reporting Summary linked to this article.

## Supplementary information

Supplementary Information

Reporting Summary

Source Data

## Data Availability

Experiment 1: Data from the main experiment and example stimuli have been deposited on the Open Science Framework: https://osf.io/h8ngp/. These data form the source data underlying all visualisations in Figs. 2a, c and 3a, c. Experiment 2: Data from the main experiment have been deposited on the Open Science Framework: https://osf.io/us87g/. These data form the source data underlying all visualisations in Figs. 2b, d and 3b, d.
