# Supplementary material for: Listeners form average-based representations of individual voice identities
Source: Nat Commun. 2019 Jun 3;10:2404. doi: 10.1038/s41467-019-10295-w (PMC6546765; doi:10.1038/s41467-019-10295-w)
Supplement: Supplementary file 1 — Supplementary Information [file 41467_2019_10295_MOESM1_ESM.pdf]

Supplementary Information

Supplementary Note 1

In this section we describe the validation process of the perceptual properties of the identities. To better understand the perceptual properties of the identities we created, we ran two pilot studies on the delexicalised stimuli created from Speaker 1. The first pilot consisted of a ratings task, assessing perceived speaker age, speaker sex and naturalness of the four different identities. The second pilot was a speaker discrimination experiment, aiming to validate that the created identities sound sufficiently distinct from each other while also including substantial within-person variability. Since the manipulations of GPR and VTL were similar across the identities created from Speaker 1 and Speaker 2, we only validated the identities created from Speaker 1 and assumed that the identities created from Speaker 2 would behave in similar ways.

We collected ratings for perceived naturalness ("How natural does this sound?" 1-not at all; 7-very much), speaker sex ("How male or female does this sound?" 1-very male; 7-very female), and speaker age ("How much does this sound like an adult?" 1-not at all; 7-very much) based on 7-point Likert scales (*N* = 15 per scale; mean age = 30.00 years, SD = 5.78 years, 23 female in total). None of the participants was familiar with the original speaker. Ratings were collected online for a subset of locations from each identity (*N* = 25; mapping the full within-person spaces via a regular 5 x 5 grid) and based on two sentences produced by Speaker 1. Ratings were similar for stimuli created from the two sentences; we therefore assumed that all sentences are perceptually similar under the manipulations. Ratings of perceived naturalness indicated that one identity (grey in Figure 1, main manuscript: low GPR, short VTL) was perceived to be the least natural-sounding identity (*M* = 2.42, *SD* = .43). Upon reviewing the stimuli, it was determined that these low ratings were likely the result of manipulation artefacts affecting the stimuli for this identity (buzzlike voice quality). This identity was thus excluded from all further tests. Ratings of speaker sex and speaker age indicated that the remaining 3 identities all sounded broadly male (Means: ID1 =3.47; ID2 = 1.56; ID3 = 3.28) and broadly adult (Means: ID1: 3.82; ID2 = 6.22; ID3 = 4.93).

We furthermore asked whether the identities are perceived as distinct from each other, and whether the within-person voice spaces big enough to be perceived as adequately variable. To this end, we ran a speaker discrimination task online on a subset of items per identity (9 items x 3 identities = 27 stimuli), again mapping the full within-person identity spaces via a regular grid. Stimuli were based on the full 69 recorded sentences. Thirty-six participants (mean age = 28.1 years, SD = 6.4 years, 19 female) who were not familiar with the original speakers took part in this study. Each participant performed a speaker discrimination task ("Same or different speaker?") on one third of all the possible pairings for the 27 voice space locations (378 pairs per participant), so that each pair was judged by 12 listeners. This pilot study showed that the 3 identities were readily perceived as distinct from one another (see Supplementary Figure 1): listeners perceived 90.5% of trials that included two nominal identities as featuring different speakers. We furthermore observed that the within-person spaces included considerable variability (e.g. [1] for a review): overall, listeners perceived 68.6% of all trials including a pair of sounds from the same nominal identity as coming from the same speaker. For some pairs sampled from within one nominal identity, listeners more often perceived items to come from two different speakers.

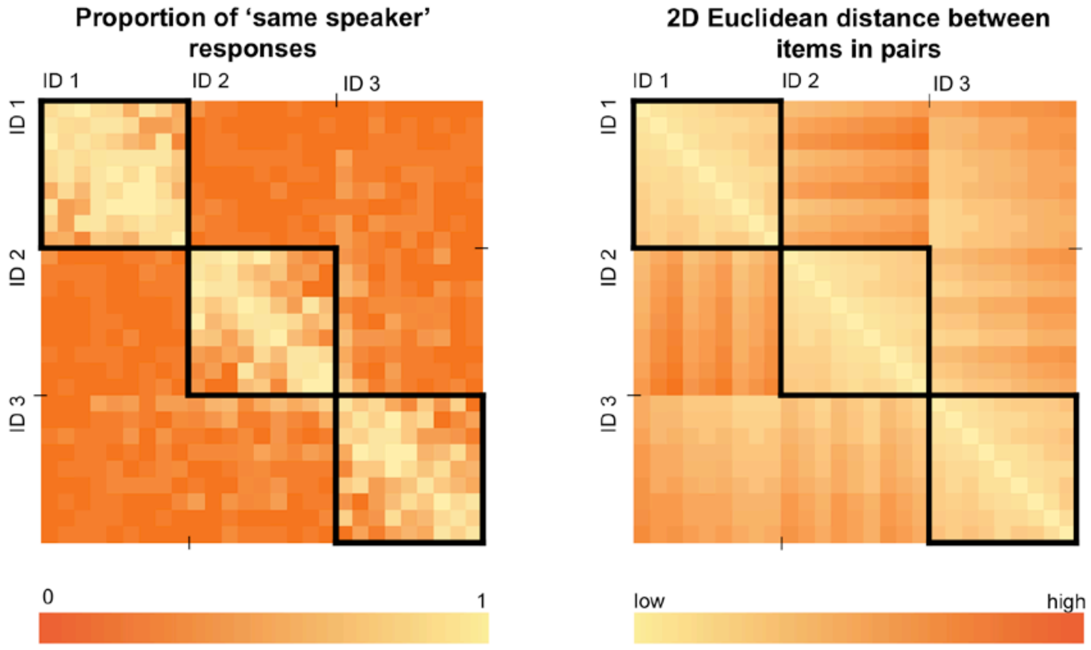

Supplementary Figure 1: Illustration of the results of the speaker discrimination pilot. The matrix on the left shows the pair-wise proportion of 'same speaker' responses, the matrix on the right shows the corresponding pair-wise 2D Euclidean acoustic distance (in GPR x VTL space) between the items within a pair. Within-person submatrices are highlighted with black borders.

Supplementary Note 2

In the main text, we reported exploratory analyses of the data for the distractor trials. We showed that accuracy (i.e. correctly labelling a distractor as “new”) was *higher* for stimuli located on the ring-shaped distribution compared to stimuli located on the centre distribution. Further, accuracy now correspondingly *decreased* the closer stimuli were to the centre (whether considering both acoustic properties together, or modelling them individually). This is the opposite pattern of results observed for the learned identities.

Before interpreting these results, it is first worthwhile to consider how listeners may have attempted to differentiate between learned and distractor identities: The existing literature on voice discrimination and voice perception has shown that GPR and VTL are used to discriminate between voices ([2]), although we note that cues used to make identity judgements for voices are likely to be at least partially voice- and listener-specific (e.g. Lavner, Gath & Rosenhouse, 2000, see also [4]). In the current experiments, GPR for the learned and distractor identities was matched precisely through manipulation, while identities also substantially overlapped substantially in the VTL dimension. By minimising the differences in GPR and VTL between the learned and distractor identities, these cues were of reduced informative value in discriminating between them. Listeners would thus have needed to use alternative or additional acoustic properties that were not explicitly matched, such as speech rate, F0 variation or periodicity features such as shimmer, jitter or HNR, in order to complete the task within the test phase of the experiments with high accuracy. However, the false alarm rates – where listeners incorrectly identified the distractor identities as “old” – were high across both experiments. This then suggests either that listeners were unsuccessful at making use of additional cues, or that they instead still relied on GPR and VTL despite these properties being largely non-diagnostic in the recognition task

It is unclear why performance differs between the centre and the training distribution for the distractor identities: these were all previously unheard and the distribution of values per se should therefore not have any impact on performance. We can propose a number of speculative explanations for these effects. First, these results may be driven by task-dependent processes: in studies that involve same/different or

match/mismatch judgements, patterns of results differ according to the trial type (e.g. [5] and [6]; Experiment 3) – this may also be the case for our recognition tests.

We may also speculate that these results could offer additional evidence for the formation of abstracted average representations during learning. First, the distractor identities fully overlap with the GPR range of the learned identities (but note that the VTL range was not fully matched). Due to this overlap, the abstracted average-based representations formed for the *learned* identities may therefore have provided a good match for the stimuli of the *distractor* identities, in particular those located in the centre distribution. Thus, distractor items at the centre yielded a greater number of false alarms, i.e. incorrect "old" responses. In contrast, distractor stimuli falling on the ring-shaped distribution were further away from the average-based representation, were a worse match to the abstracted average and were thus more readily (and accurately) recognised as a "new" identity. We note, however, that this interpretation of the results is speculative and strongly assumes that only norm-based coding underlies the formation of representations, with exemplar-based representations playing no role. It furthermore assumes ideally overlap between the distractor and learned voices. We know that this was not the case: based on our estimates (see Methods in the main manuscript) distractor and learned identities differed by around .2cm in VTL, which would correspond to a displacement of around 2 manipulation steps in our study. This difference in VTL across speakers may not, however, dramatically affect our interpretation of the results: We note that first, false alarm rates in the recognition task were high, indicating that distractor and learned identities were highly confusable. This confusability thus points at substantial perceptual overlap in perceptual properties, including VTL. Second, the relationship between accuracy and acoustic distance to the centre, while significant, is noisy. Therefore a difference in VTL may only have a limited effect, especially in the presence of matched GPR. Overall, we nonetheless stress that these interpretations of the results of exploratory analyses are highly speculative and should thus be treated accordingly.

Supplementary References

[1] Lavan, N., Burton, A. M., Scott, S. K., & McGettigan, C. Flexible voices: Identity perception from variable vocal signals. *Psychonomic Bulletin & review*, 1-13 (2018).

[2] Baumann, O., & Belin, P. Perceptual scaling of voice identity: common dimensions for different vowels and speakers. *Psychological Research 74*(1), 110-120 (2010).

[3] Lavner, Y., Rosenhouse, J., & Gath, I. The prototype model in speaker identification by human listeners. *International Journal of Speech Technology*, *4*(1), 63-74 (2001).

[4] Kreiman, J., & Sidtis, D. *Foundations of voice studies: An interdisciplinary approach to voice production and perception*. John Wiley & Sons (2011).

[5] Narayan, C. R., Mak, L., & Bialystok, E. Words get in the way: Linguistic effects on talker discrimination. *Cognitive science*, *41*(5), 1361-1376 (2017).

[6] Ritchie, K. L., & Burton, A. M. Learning faces from variability. *Quarterly Journal of Experimental Psychology*, *70*(5), 897-905 (2017).
