# Supplementary material for: Listeners form average-based representations of individual voice identities
Source: Nat Commun. 2019 Jun 3;10:2404. doi: 10.1038/s41467-019-10295-w (PMC6546765; doi:10.1038/s41467-019-10295-w)
Supplement: Supplementary file 2 — Reporting Summary [file 41467_2019_10295_MOESM2_ESM.pdf]

nature research

Corresponding author(s): Nadine Lavan, Carolyn McGettigan

Reporting Summary

Nature Research wishes to improve the reproducibility of the work that we publish. This form provides structure for consistency and transparency in reporting. For further information on Nature Research policies, see Authors & Referees and the Editorial Policy Checklist.

Statistical parameters

When statistical analyses are reported, confirm that the following items are present in the relevant location (e.g. figure legend, table legend, main text, or Methods section).

n/a	Confirmed	
☐	☒	The exact sample size (*n*) for each experimental group/condition, given as a discrete number and unit of measurement
☐	☒	An indication of whether measurements were taken from distinct samples or whether the same sample was measured repeatedly
☐	☒	The statistical test(s) used AND whether they are one- or two-sided<br>*Only common tests should be described solely by name; describe more complex techniques in the Methods section.*
☐	☒	A description of all covariates tested
☐	☒	A description of any assumptions or corrections, such as tests of normality and adjustment for multiple comparisons
☐	☒	A full description of the statistics including central tendency (e.g. means) or other basic estimates (e.g. regression coefficient) AND variation (e.g. standard deviation) or associated estimates of uncertainty (e.g. confidence intervals)
☐	☒	For null hypothesis testing, the test statistic (e.g. *F*, *t*, *r*) with confidence intervals, effect sizes, degrees of freedom and *P* value noted<br>*Give P values as exact values whenever suitable.*
☒	☐	For Bayesian analysis, information on the choice of priors and Markov chain Monte Carlo settings
☐	☒	For hierarchical and complex designs, identification of the appropriate level for tests and full reporting of outcomes
☐	☒	Estimates of effect sizes (e.g. Cohen's *d*, Pearson's *r*), indicating how they were calculated
☐	☒	Clearly defined error bars<br>*State explicitly what error bars represent (e.g. SD, SE, CI)*

Our web collection on statistics for biologists may be useful.

Software and code

Policy information about availability of computer code

Data collection	All data was collected only using the Gorilla Experiment Builder (gorilla.sc; preprint describing the platform: https://www.biorxiv.org/content/early/2018/10/13/438242).
Data analysis	Data was analysed with R (v 3.4.0) using R Studio (v 1.1.456) with the packages lme4 (v 1.1-14), psych (v 1.7.8), ggplot2 (v 3.1.0) and yarrr (v 0.1.5).

For manuscripts utilizing custom algorithms or software that are central to the research but not yet described in published literature, software must be made available to editors/reviewers upon request. We strongly encourage code deposition in a community repository (e.g. GitHub). See the Nature Research guidelines for submitting code & software for further information.

Data

Policy information about availability of data

All manuscripts must include a data availability statement. This statement should provide the following information, where applicable:

- Accession codes, unique identifiers, or web links for publicly available datasets
- A list of figures that have associated raw data
- A description of any restrictions on data availability

From the manuscript: "Experiment 1: Data from the main experiment and example stimuli have been deposited on the Open Science Framework: https://osf.io/

April 2018

h8ngp/. These data forms the source data underlying all visualisations in Figure 2a, 2c, 3a and 3c.
Experiment 2: Data from the main experiment have been deposited on the Open Science Framework: https://osf.io/us87g/. These data forms the source data underlying all visualisations in Figure 2b, 2d, 3b, 3d."

Field-specific reporting

Please select the best fit for your research. If you are not sure, read the appropriate sections before making your selection.

☐ Life sciences ☒ Behavioural & social sciences ☐ Ecological, evolutionary & environmental sciences

For a reference copy of the document with all sections, see nature.com/authors/policies/ReportingSummary-flat.pdf

Behavioural & social sciences study design

All studies must disclose on these points even when the disclosure is negative.

Study description

This a behavioural experiment on voice identity learning using human participants. All experimental data were analysed using quantitative methods (e.g. generalised linear mixed models).

Research sample

Recruitment criteria were: must be native speakers of English, aged between 18-40, have no reported hearing difficulties and must have a Prolific score of > 90 (out of 100, i.e. a high score indicating that participants provided usable data in over 90% of the studies they have previously taken part in on Prolific). Participant could furthermore not have taken part in any pilot or stimulus validation study related to this experiment. None of the participants of Experiment 2 had taken part in Experiment 1.

Sampling strategy

The sampling strategy was random using Prolific as a participant pool. The demographics of the participant pool of Prolific can be found here: https://prolific.ac/demographics/. Studies are widely advertised, and an algorithm is used to ensure that a wide range of users have a chance to sign up for studies (as opposed to a small minority of very active users signing up for most available studies).

The sample size was determined based on a power calculation to ensure > .8 power. For details, please see the preregistration document (https://osf.io/euwxk/register/565fb3678c5e4a66b5582f67). The relevant section of the preregistration has been reproduced below:

For Experiment 2, we tested 50 participants. This sample size was determined via the simr package in R allowing us to detect a similar effect size to the one reported for the confirmatory distance to centre analysis for the learned voices at at power of > .8.

Data collection

Data was collected online with the Gorilla Experiment Builder (see above). The researcher was therefore not in the room with the participants during testing. Attention checks were, however, included to ensure data quality. Participants used their own laptops or desktop computers (mobile phone and tablet users were not permitted to participate). Audio was presented via the own participants' headphones - we ensured that participants were wearing headphones via a headphone screening task (see Methods in the manuscript).

Timing

For Experiment 1, data was collected on the 7th of August 2018 with the exception of one data set, which was collected on the 8th of August. For Experiment 2, data was collected on the 13th and 14th of December.

Data exclusions

All exclusion criteria were preregistered here: https://osf.io/euwxk/register/565fb3678c5e4a66b5582f67.
Experiment 1: Out of of the 44 full data sets collected, 4 participants were excluded: 1 participant failed to give the correct response for more than 20% of vigilance trials (see Methods) and 3 participants did not perform significantly better than chance (± 95% confidence intervals) for the last 15 trials of the forced-choice recognition task of the learning phase of the experiment (see Methods in the manuscript).

Experiment 2: Out of of the 56 full data sets collected, 6 participants were excluded: all 6 participants did not perform significantly better than chance (± 95% confidence intervals) for the last 15 trials of the forced-choice recognition task of the learning phase of the experiment (see Methods in the manuscript).

Non-participation

For Experiment 1: 15 participants failed the headphones check at the start of the experiment, so they were rejected from the study before any experimental data could be collected. A further 8 participants dropped out at some stage during the study. These incomplete data sets were never viewed or taken into account for the data analysis.

For Experiment 2: 21 participants did not pass the headphones check for this experiment. A further 7 participants dropped out at some stage during the experiment (1 during training, 1 during the test phase, 5 at the consent stage).

Randomization

Stimulus sets and identity of the voices to be learned were counterbalanced across participants. This was done randomly via a counterbalancing procedure implemented on Gorilla.

Reporting for specific materials, systems and methods

Materials & experimental systems

n/a	Involved in the study	
☒	☐	Unique biological materials
☒	☐	Antibodies
☒	☐	Eukaryotic cell lines
☒	☐	Palaeontology
☒	☐	Animals and other organisms
☐	☒	Human research participants

Methods

n/a	Involved in the study	
☒	☐	ChIP-seq
☒	☐	Flow cytometry
☒	☐	MRI-based neuroimaging

Human research participants

Policy information about studies involving human research participants

Population characteristics	See "Research sample" above.
Recruitment	See also "Sampling strategy" above. Prolific generally provides a more diverse participant sample than is for example available through campus populations (broader age range, more diverse educational backgrounds, etc.). We do not anticipate that any self-selection biases have impacted the results in a predictable way.
